# Formulation and Optimization of a New Cationic Lipid-Modified PLGA Nanoparticle as Delivery System for *Mycobacterium tuberculosis* HspX/EsxS Fusion Protein: An Experimental Design

**Published:** 2019

**Authors:** Farzad Khademi, Arshid Yousefi-Avarvand, Mohammad Derakhshan, Mohammad Reza Abbaspour, Kayvan Sadri, Mohsen Tafaghodi

**Affiliations:** a *Department of Microbiology, School of Medicine, Ardabil University of Medical Sciences, Ardabil, Iran.*; b *Antimicrobial Resistance Research Center, Department of Medical Bacteriology and Virology, Qaem University Hospital, School of Medicine, Mashhad University of Medical Sciences, Mashhad, Iran. *; c *Targeted Drug Delivery Research Center, Mashhad University of Medical Sciences, Mashhad, Iran. *; d *Nuclear Medicine Research Center, Mashhad University of Medical Sciences, Mashhad, Iran* ^*. *^; e *Nanotechnology Research Center, Pharmaceutical Technology Institute, Mashhad University of Medical Sciences, Mashhad, Iran.*

**Keywords:** Mycobacterium tuberculosis, PLGA:DDA hybrid nanoparticle, HspX/EsxS fusion protein, Experimental design

## Abstract

Polymeric particles and liposomes are efficient tools to overcome the low immunogenicity of subunit vaccines. The aim of the present study was formulation and optimization of a new cationic lipid-modified PLGA nanoparticles (NPs) as a delivery system for *Mycobacterium tuberculosis* HspX/EsxS fusion protein. The cationic lipid-modified PLGA NPs containing HspX/EsxS fusion protein were prepared using a modiﬁed double emulsion solvent evaporation method. Scanning electron microscopy and dynamic light scattering (DLS) tools were used to determine physical properties of hybrid NPs. A multi-level full factorial design was used to evaluate the influence of two factors of PLGA:DDA weight ratio (w/w) and PVA concentration (%) on size, surface charge, polydispersity index, encapsulation efficiency and yield. Finally, the optimal formulation was achieved based on desired responses. Mathematical models were obtained to indicate the relation between the studied factors and responses. The DDA concentration showed an increasing effect on surface charge and also a decreasing effect on particle size, encapsulation efficiency and yield. Higher amounts of DDA increased surface charge of NPs; however, the size, encapsulation efficiency and yield were decreased. The influence of various concentrations of PVA on different physical characteristics of PLGA:DDA hybrid NPs was variable. The optimal formulation consisted of 0.91 (55:5, w/w) weight ratio of PLGA:DDA and 0.5% PVA. The hybrid NPs showed acceptable particle size distribution, strong positive surface charge, prolonged antigen release and good encapsulation efficiency in comparison to PLGA alone. However, further preclinical and clinical studies are needed.

## Introduction

*Mycobacterium tuberculosis* (*M. tuberculosis*), the causative agent of tuberculosis (TB), is one of the significant issues in global public health, due to more than two million deaths annually (1). Currently, the only efficient way for global TB control is BCG (*M. bovis* Bacillus Calmette-Guérin) vaccination. However, its protective efficacy in all parts of the worlds is not yet fully good (0 to 80%) (2). Therefore, it is an urgent need to improve or develop a new TB vaccine. Many attempts have been made to establish effective vaccines in order for either replacement with BCG, such as live recombinant BCG (rBCG) and modified non-pathogenic mycobacteria (*M. vaccae*, and *M. smegmatis*), or booster vaccines (either prophylactic or therapeutic), such as subunit vaccines, DNA vaccines, viral-vectored candidates and inactivated whole cell vaccines (2, 3). Among them, subunit vaccines are highly regarded. However, these vaccines are intrinsically weak immunogens (4). To solve this problem, polymeric nanoparticles (NPs), especially PLGA (poly (lactide-co-glycolide)) and also liposomes have attracted many attentions as efficient vaccine delivery platform/adjuvant (5-7). Both liposomes and polymeric NPs have some common benefits including adjustable size, biocompatibility and high encapsulation efficiency (8). An important feature of liposomes is the ability to encapsulate both hydrophilic and hydrophobic materials in their aqueous core or between lipid layers, respectively (8). PLGA is a non-toxic, biocompatible and biodegradable synthetic polymer approved by the U.S Food and Drug Administration (FDA) for human application and the proteins and peptides nanomedicines, etc. (9, 10). It has been used to develop the drug delivery and vaccine delivery systems due to controlled release properties, versatile degradation to completely safe materials *in-vivo*, easy production and structural diversity (10). In recent years, various studies have demonstrated that the surface of PLGA could be coated with cationic lipids such as DOTAP, DOPC, and DOPG in order to form lipid-polymer complexes, hybrid NPs, which can be an excellent TB vaccine delivery platform with high rate of antigen encapsulation, prolonged release of antigen, good serum stability and induced strong immune responses (9-12). DDA, dimethyl dioctadecylammonium bromide, is a cationic liposome-forming lipid classified in quaternary ammonium compounds. This synthetic amphiphilic lipid in combination with different immunomodulators is well defined as an excellent adjuvant in order to promote humoral and Th1 type of cell-mediate responses (13). The aim of this study was to design a new cationic lipid-modified PLGA hybrid NP as an excellent delivery system for *M. tuberculosis* HspX/EsxS fusion protein.

## Experimental

*Materials*

PLGA (Resomer® RG 752 H, poly (D, L-lactide-co-glycolide) acid terminated, lactide:glycolide 75:25, MW 4,000-15,000), PVA (poly (vinyl alcohol), MW 89000 to 98000, 99% degree of hydrolysis) and DDA were purchased from Sigma-Aldrich Chemie GmbH (Germany). Dichloromethane at analytical grade was obtained from Sigma-Aldrich.

**Table 1:** Factorial design parameters: independent variables and levels

**Independent variables**	**Levels**				
X1: PLGA:DDA weight ratio (w/w)	30:30	40:20 (0.66)	50:10 (0.83)	55:5 (0.91)	100:0 (1)
X2: PVA concentration (%)		0.5	1	2	

**Table 2:** Physical characteristics of PLGA:DDA hybrid NPs

**Test run**	**PLGA ratio in NPs**	**PLGA** ** (mg)**	**DDA (mg)**	**PVA concentration (%)**	**Z-average (nm)**	**Zeta-potential (mV)**	**PDI**	**Encapsulation rate** **(%)**	**Yield** **(%)**
1	0.5	30	30	0.5	261.2±100.8	33.4±7.83	0.307±0.04	8.2±0.6	23±2.6
2	0.66	40	20	0.5	230.4±12.6	40.7±1.7	0.289±0.08	14.1±2.2	29.6±1.5
3	0.83	50	10	0.5	249.7±16.7	39±1.8	0.233±0.07	35.7±1.4	41±2
4	0.91	55	5	0.5	228.7±15.1	39.1±3.5	0.229±0.04	63±1.9	46.3±1.5
5	1	60	0	0.5	316.7±12.7	-33±1.7	0.218±0.03	92.2±2	50.1±2.1
6	0.5	30	30	1	200.7±26.5	35.1±3.36	0.289±0.08	10±1	27.3±2.5
7	0.66	40	20	1	202.9±22.5	32.8±8.8	0.217±0.04	16±1	32.6±3.2
8	0.83	50	10	1	219.7±24.8	32.26±4.1	0.229±0.13	41.4±7	42.6±2.5
9	0.91	55	5	1	351.2±39.6	8.7±5.92	0.252±0.12	70.6±2	45.6±3
10	1	60	0	1	360.1±31.3	-26.6±2.7	0.233±0.07	94.1±2	49.4±1
11	0.5	30	30	2	213.2±16.1	31.8±2.45	0.189±0.06	9.5±2.4	26±2
12	0.66	40	20	2	280.9±5.9	32.7±1.	0.244±0.03	22.5±2.4	31.3±1.5
13	0.83	50	10	2	288.5±5.8	32.5±4.2	0.230±0.07	49.2±13.9	41.3±1.5
14	0.91	55	5	2	317.9±1.2	5.52±6.3	0.472±0.1	83.4±5.5	45.3±2.5
15	1	60	0	2	319.7±4.3	-31.5±9.2	0.312±0.05	91.2±6	48±1

All data presented as means ± SD (n = 3).

**Figure 1:**
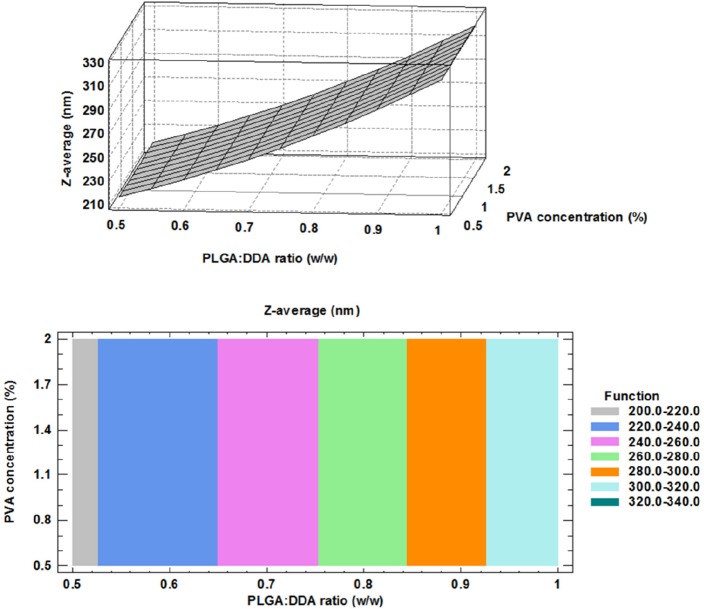
Effect of formulation factors on particle size (Z-average); above: 3D surface plot, below: contour plot

Z-average (nm): 181.832 + 137.848 X 2, R2= 0.348             (1)

**Table:** 

Model	Sum of Squares	df	Mean Square	F	Sig.*
Regression	61780.614	1	61780.614	22.984	0.001
Residual	115583.514	43	2687.989		
Total	177364.128	44			

Predictors: (Constant), X1X1

Dependent Variable: Z-average (nm)

*Level of signiﬁcance *p*< 0.05

**Figure 2:**
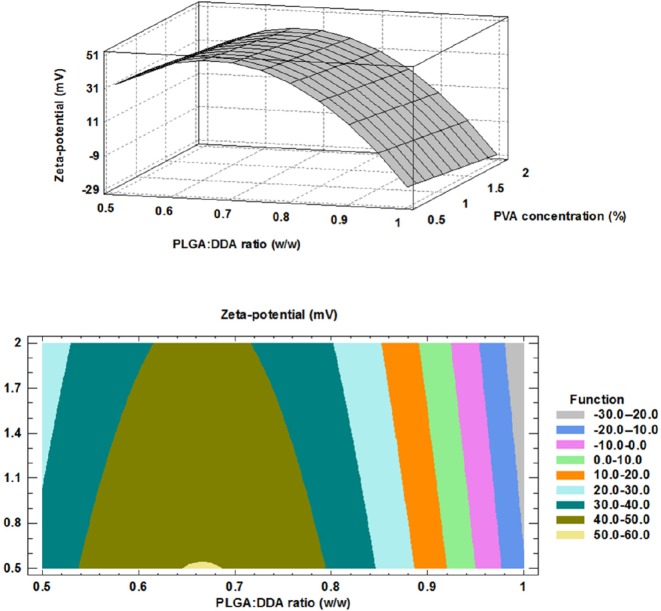
Effect of formulation factors on Zeta-potential (mV); above: 3D surface plot, below: contour plot

Zeta-potential (mV): -223.506 + 830.920 X - 5.790 X - 623.854 X 2, R2= 0.842             (2)

**Table:** 

Model	Sum of Squares	df	Mean Square	F	Sig.*
Regression	26153.888	3	8717.963	72.856	0.0001
Residual	4906.054	41	119.660		
Total	31059.942	44			

Predictors: (Constant), X1X1, X2, X1 Dependent Variable: Zeta-potential (mV)

*Level of signiﬁcance *p*< 0.05

**Figure 3:**
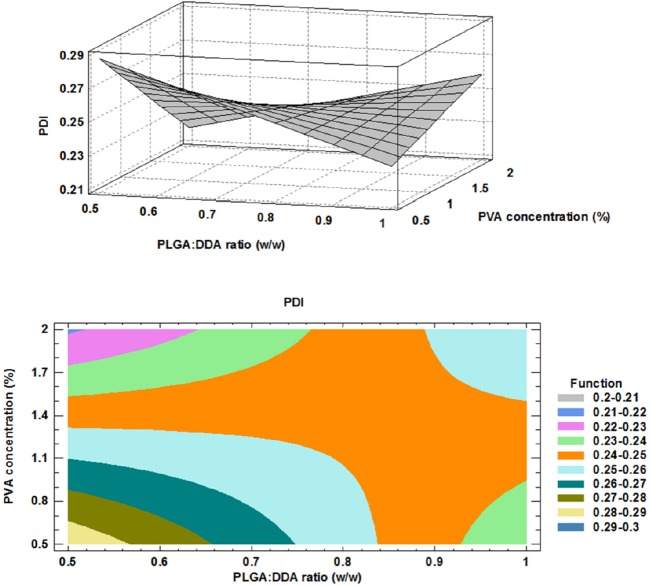
Effect of formulation factors on polydispersity index; above: 3D surface plot, below: contour plot

PDI: 0.398 - 0.175 X - 0.110 X + 0.128 X X , R2= 0.471             (3)

**Table:** 

Model	Sum of Squares	df	Mean Square	F	Sig.*
Regression	.170	3	.057	12.178	0.0001
Residual	.191	41	.005		
Total	.361	44			

Predictors: (Constant), X1X2, X2, X1 Dependent Variable: PDI

*Level of signiﬁcance *p*< 0.05

**Figure 4:**
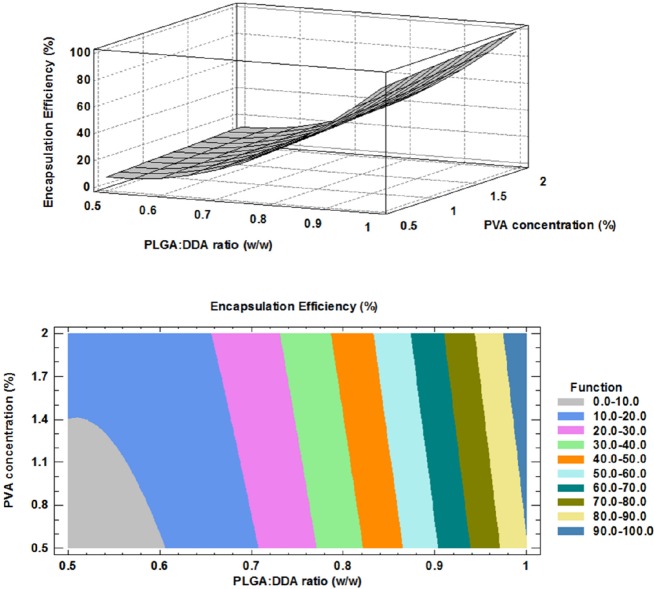
Effect of formulation factors on encapsulation efficiency (%); above: 3D surface plot, below: contour plot

Encapsulation efficiency (%): 101.362 – 367.654 X + 352.825 X 2 + 6.068 X X , R2= 0.977             (4)

**Table:** 

Model	Sum of Squares	df	Mean Square	F	Sig.*
Regression	45847.018	3	15282.339	584.214	0.001
Residual	1072.511	41	26.159		
Total	46919.530	44			

Predictors: (Constant), X1X2, X1X1, X1

Dependent Variable: Encapsulation Efficiency (%)

*Level of signiﬁcance *p*< 0.05

**Figure 5:**
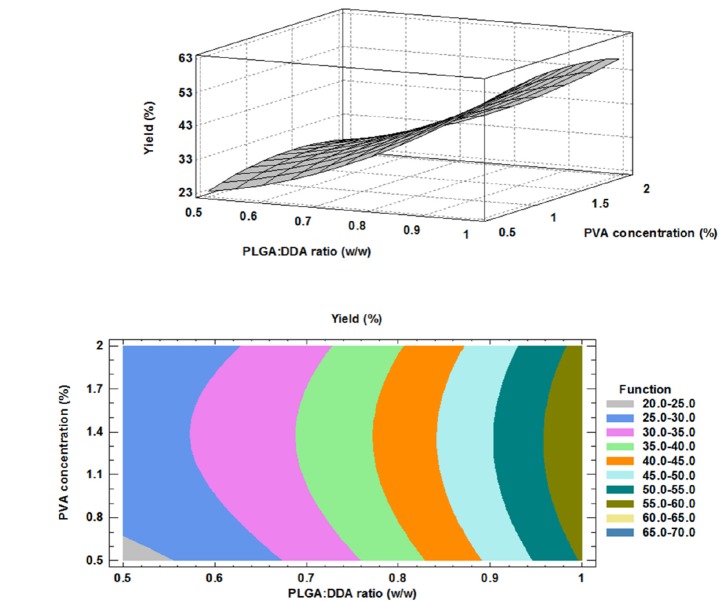
Effect of formulation factors on yield (%); above: 3D surface plot, below: contour plot

Yield (%): 24.634 - 55.175 X + 16.380 X + 79.903 X 2 - 5.568 X 2 - 1.592 X X , R2= 0.977            (5)

**Table:** 

Model	Sum of Squares	df	Mean Square	F	Sig.*
Regression	5767.418	5	1153.484	338.189	0.001
Residual	133.020	39	3.411		
Total	5900.438	44			

Predictors: (Constant), X1X2, X1X1, X2X2, X2, X1 Dependent Variable: Yield (%)

*Level of signiﬁcance *p*< 0.05

**Figure 6:**
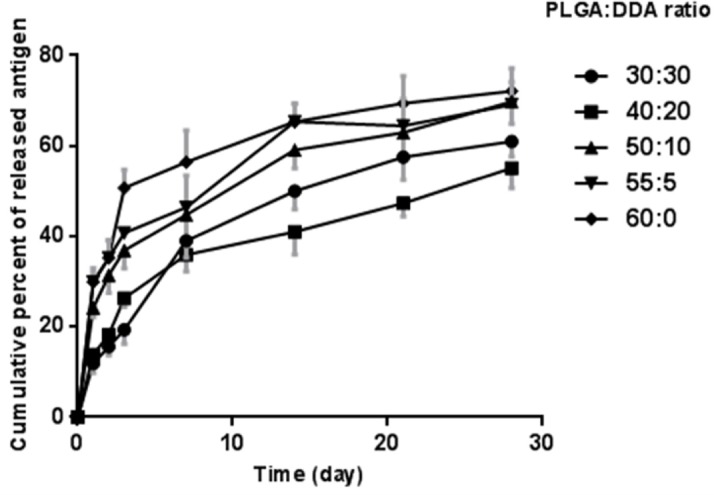
Cumulative release of HspX/EsxS fusion protein from different formulation of PLGA:DDA hybrid NPs in PBS (pH 7.4) at 37°C. All data presented as means ± SD (n = 3)

**Figure 7:**
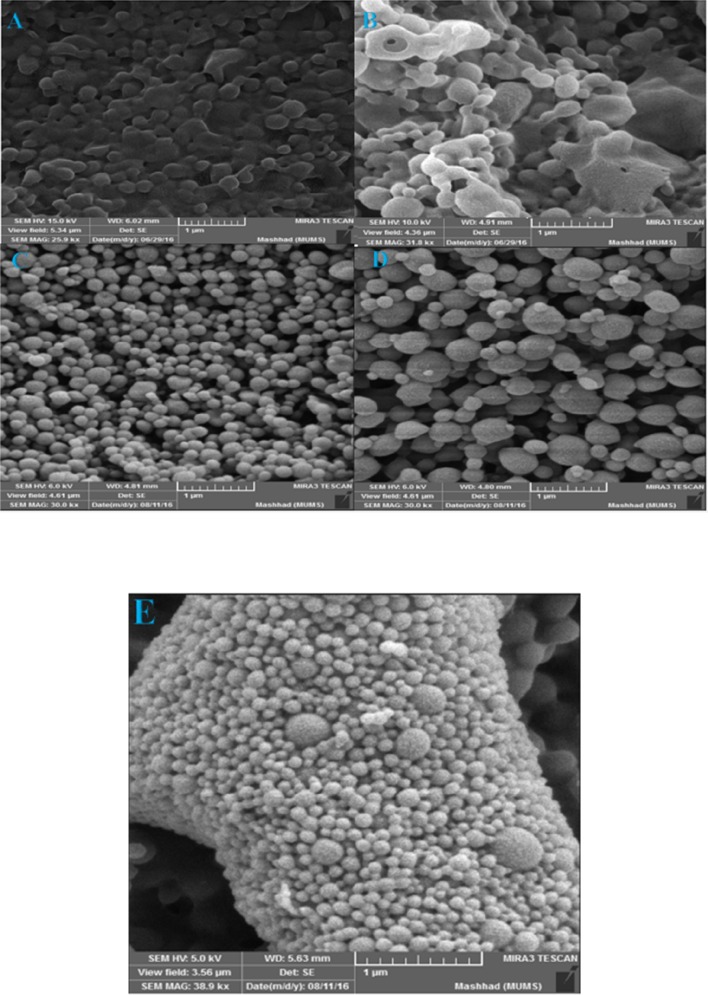
Scanning electron micrograph of surface of PLGA:DDA hybrid NPs containing 30 mg PLGA and 30 mg DDA (A), 40 mg PLGA and 20 mg DDA (B), 50 mg PLGA and 10 mg DDA (C), 55 mg PLGA and 5 mg DDA (D) and 60 mg PLGA and 0 mg DDA (E). (Scale bar represents 1 µm)

**Figure 8:**
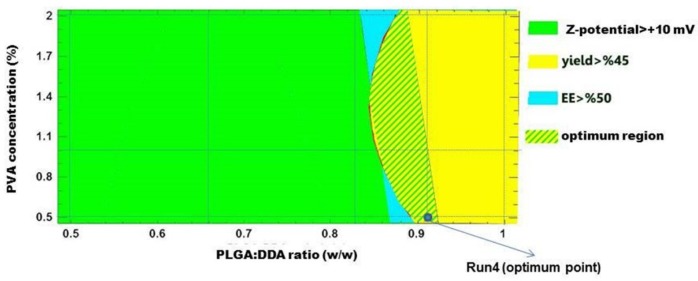
Display of superimposed contour plot to identify the optimal formulation; Run4 is selected as an optimum point

*Experimental design*

A multi-level full factorial design was used to formulate the hybrid NPs by double emulsion solvent evaporation method. The effect of two parameters of PLGA:DDA weight ratio (w/w) in 5 levels and PVA concentration (%) in 3 levels, as independent variables, were evaluated. The design parameters and their levels are shown in Table 1. 

The impact of these parameters on physical properties of hybrid NPs such as mean particle diameter (Z-average, nm), surface charge (Zeta-potential, mV), polydispersity index (PDI), encapsulation efficiency (%) and yield (%), as dependent variables or responses, were studied (Table 1).

*Preparation of PLGA:DDA hybrid NPs*

Molecular cloning, expression and purification of* M. tuberculosis* HspX/EsxS fusion protein was previously performed and reported (14). PLGA:DDA hybrid NPs were prepared using a modiﬁed w/o/w double emulsion solvent evaporation method (9). Brieﬂy, as shown in Table 2, various weight ratios of PLGA and DDA, at a final concentration of 60 mg/mL, were weighted and dissolved in 600 µL dichloromethane as organic phase. Hundred twenty µL of a 1 mg/mL HspX/EsxS fusion protein solution in ultrapure water was added to organic phase. The first sonication was performed by an ultrasonic homogenizer (Hielscher – Ultrasound Technology, Germany) for 30 s to establish water-in-oil (w_1_-o) emulsion. To obtain water-in-oil-in-water (w_1_-o-w_2_) emulsion, 4 mL of 0.5, 1, and 2% (w/v) of PVA was added to w_1_-o emulsion and then second sonication was performed for 60 s. To complete evaporation of dichloromethane, the suspension was slowly added to 20 mL of 0.3% (w/v) of PVA and stirred for 24 h. Finally, the suspension was centrifuged at 18000 *g*, 4 °C for 12 min and the pellet was washed three times with 10 mL of ultrapure water. NPs were re-suspended in 1 mL ultrapure water, freeze-dried and stored at 4 °C for later use.

*Evaluation of physical characteristics of PLGA:DDA hybrid NPs*

Complete physical characteristics of hybrid NPs including Z-average (nm), Zeta-potential (mV) and PDI were determined by dynamic light scattering (Zetasizer Nano, Malvern, UK). For above purposes, 1 mg of each freeze-dried NP formulation was re-suspended in 1 mL of ultrapure water and sonicated in an ultrasonic bath for 2 min. The scanning electron microscopy (SEM) was used to evaluate the morphology of protein-loaded hybrid NPs. NPs yield was determined as the percentage of residual NPs to the theoretical weight after freeze-drying.

*Determination of HspX/EsxS encapsulation efﬁciency*

In order to determine the encapsulation efﬁciency of HspX/EsxS fusion protein, this protein was first radiolabelled by an iodination technique (15). Briefly, 10 µg of purified HspX/EsxS protein was diluted with 45 µL of phosphate buffer (0.5 M, pH 7.5) and added to 10 µL of predetermined activity of ^125^I (200 µCi). Then, 20 µL of chloramine-T (2 mg/mL, pH 7.5), as a powerful oxidizing agent, and 50 µL of sodium metabisulfite (Na_2_S_2_O_5_) (4 mg/mL, pH 7.5), as a reducing agent, were added and the mixture stirred for 30 s and 60 s at room temperature, respectively. The reaction mixture was further diluted with 100 µL of potassium iodide (KI) (10 mg/mL, pH 7.5) and stirred for 2 min and then purified by the gel filtration chromatography column. Pooled protein aliquots were used for preparation of PLGA:DDA hybrid NPs to determine encapsulation efﬁciency. Protein encapsulation efﬁciency was determined by Gamma counter (Packard Instruments Company Inc., IL, USA) using the following equation: Protein encapsulation efﬁciency (%) = Gamma radiation emitted from pellet/Total gamma radiation × 100 (16). Thin layer chromatography (TLC) technique using silica gel, as the stationary phase, and normal saline, as the mobile phase, Were used to evaluate radiolabelling efficiency and physical stability of HspX/EsxS protein-NP complex in human serum and PBS medium.

*In-vitro HspX/EsxS protein release proﬁle*

To evaluate the release proﬁle, ^125^I-labelled HspX/EsxS protein was encapsulated with PLGA:DDA hybrid NPs. NPs were suspended in a 15 mL release medium (PBS, pH 7.4) and incubated at 37 °C under constant agitation. At certain time points, 1 mL samples of medium were taken and centrifuged at 10000 *g* for 15 min. Gamma radiation emitted from supernatant was counted and recorded. Followed equation was used to calculate the protein release proﬁle, HspX/EsxS release percent = Gamma radiation emitted from supernatant/Total gamma radiation × 100. Finally, 1 mL of fresh buffer was added to release medium (16).

*Data analysis*

By using a polynomial equation: Y = a_1_X_1_ + a_2_X_2 _+ a_3_X_1_^2^ + a_4_X_2_^2^ + a_5_X_1_X_2_, the influence of independent variables on dependent variables were evaluated. Statistical analysis of data and the modeling were performed through the SPSS software (version 16). Statgraphics Centurion software (version 16) was used to draw the surface plot and the contour plot. All experiments were repeated for 3 times and the results expressed as mean ± SD and if *p *< 0.05, it was considered as statistically 

significant.

## Results and Discussion

*Effect of formulation factors on particle size distribution*

PLGA:DDA hybrid NPs were produced by the modiﬁed w/o/w double emulsion solvent evaporation method with different levels of two parameters, PLGA:DDA weight ratio (w/w) and PVA concentration (%). Physical characteristics of PLGA:DDA hybrid NPs including particle size, Zeta-potential, PDI, encapsulation efficiency, yield and composition of different formulation were listed in Table 2. A key factor for the adjuvant activity of PLGA NPs is their particle size. Particle sizes in the range of 300 to 600 nm are capable to enhance type 1 (Th1) immune responses due to more efficient uptake by antigen-presenting cells (APCs), which is necessary for effective TB immunity, while in the range of 2-8 µm, they induce Th2 responses (17). Except for 0.5% PVA, there is a decrease in the size distribution from 360.1 ± 31.3 nm at 0% DDA (w/w) to 200.7 ± 26.5 nm at 50% DDA (w/w) for 1% PVA concentration as well as from 319.2 ± 4.3 nm to 213.2 ± 16.1 nm for 2% PVA concentration (Table 2). 

The correlation between Z-average (nm), PLGA:DDA weight ratio and different concentrations of PVA were clearly shown in the three dimensional display of surface plot and contour plot (Figure 1). An obvious positive effect of the DDA weight ratio to total concentration of hybrid NPs was observed for the particle size. Based on Figure 1, a significant concentration-dependent decrease in the particle size (nm) was observed by increasing the concentration of cationic lipid DDA from 0% to 50% (w/w) (*p *< 0.05). Similar results in agreement with this study have been shown by using of other cationic compounds like DOTAP and PEI to modify the PLGA NPs (9). However, Kirby and colleagues reported that the addition of the DDA to the NPs formulation led to increase in the size due to aggregation (16). Increasing PVA concentration from 0.5% to 2% had no effect on the mean particle size (Figure 1). The obtained model for particle size and the results of regression analysis are as follow:

*Effect of formulation factors on surface charge*

It has been established that increase in surface charge of NPs (Zeta-potential, mV) has a positive impact on induction of strong immune responses (16). Furthermore, positively charged NPs show more interaction and cellular uptake and also allow for more antigen adsorption (17, 18). Based on Hu and colleagues study, PLGA NPs modified with cationic lipid showed more stability, more prolonged *in-vitro* antigen release and better uptake by dendritic cells (DCs) (8). As compared with negatively charged and neutral particles, positively charged particles show more interaction and uptake into the cells as well as escape from the lysosomes. This is performed through the ionic interaction with negatively charged cell membranes (18-20). At the present study, negatively charged PLGA polymer was selected as the backbone of a hybrid NPs and their surface were modified with cationic lipid, DDA. After addition of DDA, total surface charge changed to neutral or positive. 

The impact of PLGA:DDA weight ratio (w/w) and PVA concentration (%) on surface charge have been shown in Figure 2. By increase in DDA weight ratio from 0 to 50% (w/w), Zeta-potential (mV) showed a nearly same positive trend in all three PVA concentrations and surface charge of NPs was changed from negative for unmodified PLGA NPs to positive for modified type (*p *< 0.05) (Table 2). Positively charged DDA electrostatically interacts with negatively charged PLGA via its quaternary ammonium compounds and changes physicochemical characteristics of hybrid NPs (17). The influence of PVA concentration on the surface charge of NPs was negligible. 

The obtained model for surface charge and the results of regression analysis are given below:

*Effect of formulation factors on PDI*

The polydispersity index (PDI) represents the width of the size distribution and is a measure for the heterogeneity of particle sizes. The PDI values are in the range of 0 to 1. Uniform or monodisperse particles show 0 value (10). As shown in Figure 3, the influence of DDA addition to the formulations was variable in the different PVA concentrations. In the case of 2% PVA concentration, by increasing of DDA concentration, more monodisperse NPs was obtained and PDI was changed from about 0.4 to about 0.1. However, in 0.5% PVA concentration, PDI increased from about 0.2 to about 0.3 nm, a non-uniform (polydisperse) formulation which has an inconsistent size. However, in 1% PVA concentration, changes in DDA concentration have no effect on PDI. The obtained model for PDI and the results of regression are given below: 

*Effect of formulation factors on encapsulation efficiency*

One of the most important characteristics of PLGA NPs as an ideal candidate for the delivery of the subunit vaccines is prolonged release of encapsulated antigens which is important to eliminate or reduce multiple booster doses of subunit vaccines (21). Encapsulation of the subunit vaccines with NPs could improve their *in-vitro *and* in-vivo *physical stability and prevent from changes in the protein structures such as protein denaturation and aggregation as well as chemical instability such as hydrolysis, oxidation and deamination (21). 

The HspX/EsxS fusion protein was encapsulated with PLGA:DDA hybrid NPs with varying amounts of DDA. As shown in Table 2 and Figure 4, in all formulations, DDA has a negative effect on encapsulation rate of HspX/EsxS fusion protein. Increase in weight ratio of DDA from 0 to 50% (w/w) led to a significant decrease in antigen entrapment efficiency (*p *< 0.05). This observation can be attributed to more porosity of PLGA:DDA NPs with more DDA ratio and resulting leakage of encapsulated HspX/EsxS (16). Similar to the surface charge response, the influence of various PVA concentrations on the encapsulation efficiency was negligible. The obtained model for encapsulation efficiency and the results of regression are given below:

*Effect of formulation factors on NPs yield*

The higher production yield of NPs means the lower production costs. Therefore, evaluation of different factors on NPs production yield is essential. As shown in Table 2, in all of PVA concentrations, more PLGA:DDA ratios resulted higher production yields (23 ± 2.6 to 55.1 ± 2.1 mg, 27.3 ± 2.5 to 58.4 ± 1 mg and 26 ± 2 to 57 ± 1 mg at 0.5%, 1% and 2% PVA concentrations, respectively). Murakami and colleagues have also showed that the appropriate selection of organic solvents could optimize the NPs yield (22). Here, effects of two formulation factors on NPs yield were studied. 

The PVA concentration had no significant effect on the yield. However, as shown in Figure 5, DDA concentration had a negative effect on NP yield (*p *< 0.05). Like encapsulation efficiency model, the obtained model for PDI was accurate and model equation and the results of regression of responses are given below:

*Effect of formulation factors on antigen release profile*

*In-vitro* release characteristics of *M. tuberculosis *HspX/EsxS fusion protein from various formulations of lipid-modified PLGA NPs were studied in a 15 mL release medium (PBS, pH 7.4) for 1 month (Figure 6). As shown in Figure 6, after 1 day, an initial release with less than 20% for 30:30 (w/w) and 40:20 (w/w) weight ratios and more than 20% for 50:10 (w/w) and 55:5 (w/w) weight ratios as well as unmodified PLGA NPs was observed. 

The hybrid NPs with 30:30 (w/w) ratio displayed the least antigen release rate up to the first 3 days, however, from day 3 to 28, 40:20 (w/w) ratio of the hybrid NPs showed the least antigen release rate. In comparison with cationic lipid-modified PLGA with a sustained and prolonged release profile, unmodified PLGA NPs showed the most rapid antigen release.

*Morphology of PLGA:DDA hybrid NPs*

The morphology of hybrid NPs was evaluated by scanning electron microscopy (SEM) (MIRA3 LM, Czech Republic). For this purpose, an amount of freeze-dried NPs were prepared on aluminum stubs using double-sided carbon tape and then by using sputter coater (Quorum Technologies Ltd, UK) and under Argon atmosphere, a thin film of Gold particles were sputter coated on NPs. As shown in Figure 7, increasing the amount of DDA leads to the agglomeration and aggregation of particles and makes the irregular shapes. In blank PLGA and also NPs with low concentration of DDA, the shape of NPs was spherical and with smooth surface. However, it was irregular in higher concentration of DDA. There was a difference between the size distributions measured by SEM and DLS.

*Optimization of NPs formulation*

To identify the optimal formulation, following criteria as the desired range of each parameter was selected: Zeta-potential > +10 mV, yield > 45% and encapsulation efficiency > 50%. A graphical approach was used by superimposing the contour plots of mentioned responses to obtain the optimum region and an optimum point for hybrid NPs formulation. As shown in Figure 8, the optimal formulation was formulation No. 4 which consisted of 91% or 55:5 (w/w) weight ratio of PLGA:DDA and 0.5% PVA. 

## Conclusion

Production of a new cationic lipid-modified PLGA NP as delivery system for *M. tuberculosis* HspX/EsxS fusion protein was reported. Hybrid NPs showed desirable particle size distribution and strong positive surface charge, due to adding DDA, which could be adsorbed by APCs more efficiently and show a strong induction of Th1 type of cell-mediate responses. Hybrid NPs showed also prolonged antigen release and acceptable encapsulation efficiency which can be use be used to eliminate or reduce multiple booster doses of future subunit vaccines. We are optimistic about results obtained in order to introduction of PLGA:DDA NPs as a potential antigen delivery system. However, to confirm these claims and whether the vaccine can induce innate and adaptive immune responses after challenge and reduce the bacterial load and bacterial growth in early TB exposed individuals, further preclinical and clinical studies are needed.
